# Characteristics and correlates of electronic cigarette product attributes and undesirable events during e-cigarette use in six countries of the EUREST-PLUS ITC Europe Surveys

**DOI:** 10.18332/tid/93545

**Published:** 2018-08-05

**Authors:** Christina N. Kyriakos, Filippos T. Filippidis, Sara Hitchman, Charis Girvalaki, Chara Tzavara, Tibor Demjén, Esteve Fernandez, Ute Mons, Antigona Trofor, Yannis Tountas, Mateusz Zatoński, Witold A. Zatoński, Geoffrey T. Fong, Constantine I. Vardavas, on behalf of the EUREST-PLUS consortium

**Affiliations:** 1 European Network on Smoking and Tobacco Prevention (ENSP), Brussels, Belgium; 2 University of Crete (UoC), Heraklion, Greece; 3 University of Athens (UoA), Athens, Greece; 4 King’s College London (KCL), London, United Kingdom; 5 Smoking or Health Hungarian Foundation (SHHF), Budapest, Hungary; 6 Institut Català d’Oncologia (ICO) and Bellvitge Biomedical Research Institute (IDIBELL), Catalonia, Spain; 7 School of Medicine and Health Sciences, Universitat de Barcelona, Catalonia, Spain; 8 Cancer Prevention Unit and WHO Collaborating Centre for Tobacco Control, German Cancer Research Center (DKFZ), Heidelberg, Germany; 9 University of Medicine and Pharmacy ‘Grigore T.Popa’, Iasi, Romania; 10 Aer Pur Romania, Bucharest, Romania; 11 Health Promotion Foundation (HPF), Warsaw, Poland; 12 London School of Hygiene and Tropical Medicine, London, United Kingdom; 13 University of Waterloo (UW), Waterloo, Canada; 14 Ontario Institute for Cancer Research, Toronto, Canada

**Keywords:** cross-sectional study, electronic cigarette, e-cigarettes, tobacco products directive, regulatory science

## Abstract

**INTRODUCTION:**

This study assessed characteristics and correlates associated with e-cigarette product attributes and identified correlates of experiencing undesirable events during e-cigarette use among adult smokers across six European Union (EU) Members States (MS) prior to the implementation of the Tobacco Products Directive (TPD) in 2016.

**METHODS:**

We conducted a cross-sectional survey with a nationally representative sample of adult cigarette smokers from six EU MS (Germany, Greece, Hungary, Poland, Romania, Spain) reporting e-cigarette use; randomly selected through a multistage cluster sampling design from June to September 2016. Stepwise logistic regressions were used to identify factors associated with use of flavors, noticing health warnings, mixing e-liquids, experiencing ‘dry puff’, e-liquid leaking during use and e-liquid spilling during refill.

**RESULTS:**

Current daily or weekly prevalence of e-cigarette use among this sample of adult smokers was 7.5%. The most common attributes of e-cigarettes used included those that are flavored, contain nicotine, and are of tank style. Noticing health warnings on e-cigarette packaging and leaflets, respectively, was low (10.2% and 28%, respectively). Use of e-liquid refill nozzle caps, described as easy for a child to open, was associated with spilling during refill (OR=6.73; 95% CI: 2.02–22.37). Participants who adjusted occasionally or regularly the power (voltage) or temperature of their e-cigarette had greater odds of ever experiencing a ‘dry puff’ (OR=6.01; 95% CI: 2.68–13.46). Mixing different e-liquids was associated with leaking during use (OR=7.78; 95% CI: 2.45–24.73) and spilling during refill (OR=8.54; 95% CI: 2.29–31.88).

**CONCLUSIONS:**

Ongoing evaluation of factors associated with e-cigarette attributes and of the correlates of experiencing e-cigarette undesirable events during use, related to product design, is crucial to monitoring the impact of the implementing Acts of the EU TPD.

## INTRODUCTION

Electronic cigarette (e-cigarette) use and experimentation has increased in recent years across the European Union (EU), with rates being highest among cigarette smokers^1,2^. Uncertainty around the potential impact of e-cigarettes on health, their role in smoking cessation, and their uptake among nonsmokers and youth has provoked much debate, while also propagating research that has contributed to an evolving evidence base whose contribution can only be fully ascertained with time^3–8^. Notwithstanding these issues, as a consumer product, e-cigarettes have prompted a call for regulation by the European Commission (EC) to ensure a level of consumer safety and awareness of the risks for its users. The EU Tobacco Products Directive (TPD) 2014/40/EU^9^, which aims to mitigate the burden of tobacco morbidity and mortality in the EU through tobacco product regulation, along with Commission Implementing Decisions EU 2016/586 (2016)^10^ and EU 2015/2183 (2015)^11^, establishes standards for e-cigarette product safety, packaging, and reporting. Specifically, the provisions of EU TPD Article 20 enumerate product design requirements including, but not limited to, refill container volume, nicotine content levels, child-resistant packaging features, health warning labels, informational leaflets, and technical parameters to reduce the risk of spilling during refill or leaking during use^9^. These requirements were developed with the aim to mitigate four main risks associated with use of e-cigarettes identified by the EC in Commission Report COM (2016) 269 including: ‘(1) poisoning from ingesting e-liquids containing nicotine (especially for young children), (2) skin reactions related to dermal contact with e-liquids containing nicotine and other skin irritants, (3) risks associated with home blending and (4) risks due to using untested combinations of e-liquid and device or hardware customization’^12^.

Based on the scientific literature^13^, it is the underlying assumption that the EU TPD Article 20 regulatory requirements, which effectively provide specifications on ‘product attributes’, will protect people from the aforementioned risks by reducing the occurrence of ‘undesirable events’ during product use. For instance, in light of the evidence on the toxicity of nicotine containing liquids, the EU TPD provision on compliant refill mechanisms is designed to minimize the risk of poisoning from accidental ingestion or skin reactions from dermal contact by reducing the occurrence of e-liquid leaking during use or spilling during refill^12^. An additional risk mitigation strategy of EU TPD Article 20 includes requirements of a leaflet with instructions on proper use during refill and consumption, as well as of appropriate health warning labels to communicate health risks to consumers^9^.

Additional e-cigarette product attributes, which could result in undue risk exposure, may be beyond what are currently regulated in the EU TPD. The EC has proposed that attributes that warrant further research include, but not limited to, characterizing flavors and product features that allow user customization (i.e. mixing e-liquids and adjusting voltage or temperature settings)^12^. For example, there are concerns that incorrect^12^ modifications to voltage or temperature settings on e-cigarette devices may increase exposure to non-nicotine toxicants, with particular concern during ‘dry puff’ conditions^14,15^. Home blending or mixing of e-liquids can also circumvent quality control measures that may result in increased exposure to high concentrations of nicotine and to inappropriate ingredients and ratios of Propylene Glycol/Vegetal Glycerin (PG/VG)^12,13^. Furthermore, the variety of flavors used in many e-cigarettes raises issues of allurement and product initiation^16^ and justifies research on the toxicological and health hazard profiles of flavoring additives^12,17^.

In light of the need to evaluate the impact of the EU TPD Article 20, coupled with the EC’s call to examine additional product attributes of e-cigarettes, the aims of the current study were to identify the characteristics and factors associated with e-cigarette product attributes and to examine correlates of experiencing e-cigarette undesirable events during use, as well as noticing health warnings and leaflets, among adult smokers who also use e-cigarettes, across six EU Member States (Germany, Greece, Hungary, Poland, Romania, Spain). Given that consumer use of e-cigarettes may change, as the products evolve under regulatory and market influence, it is additionally crucial to monitor how these products are being used over time, particularly under the implementation of the EU TPD. Within the global regulatory context of e-cigarettes, the EU TPD is one of the most advanced regulations with regards to e-cigarette product regulation and packaging^9,18^, and therefore an understanding of its impact has vast regulatory research and policy implications beyond the EU.

## METHODS

### Design

The current study is part of an EC Horizon 2020 funded project entitled **European Regulatory Science on Tobacco: Policy implementation to reduce lung diseases (EUREST-PLUS-HCO-06-2015)**. The overall objective of the EUREST-PLUS H2020 project is to monitor and evaluate the impact of tobacco control policies at a European level within the context of the newly implemented TPD and the World Health Organization (WHO) Framework Convention on Tobacco Control (FCTC)^19^. A major aim of EUREST-PLUS is to evaluate the psychosocial and behavioural impacts of the TPD and WHO FCTC implementation, through the creation of a cohort study of adult smokers in six EU MS in a preTPD vs postTPD study design. The methodology of the International Tobacco Control Policy Evaluation (ITC) Project, was applied and provided the theoretical framework and methods to evaluate the impact of key provisions of the FCTC and TPD within these six EU MS.

This baseline EUREST-PLUS ITC Wave 1 Survey was conducted with a nationally representative sample (total N=6011) of adult cigarette smokers (≥18 years) from six EU MS between June and September 2016 prior to the full implementation of the provisions of Article 20 of the TPD^9^ and its respective Commission Implementing Decisions^10,11^. Participants in each country were recruited using a multistage cluster sampling design, with geographical strata based on the Nomenclature of Territorial Units for Statistics (NUTS), with regards to degree of urbanization (urban, intermediate, rural). Using a random walk design, household addresses were sampled from 100 clusters per country, with approximately 10 adult smokers enrolled per cluster. From each household, one male and one female smoker were randomly selected to complete an interview. Interviews were conducted face-to-face by interviewers using tablets (CAPI). Additional details on the EUREST-PLUS ITC Survey Wave 1 are described elsewhere^20^.

### Measures

The conceptual model of all ITC Surveys, which is based in psychosocial behavioural theories^19^, guided the selection of questions in the EUREST-PLUS ITC Wave 1 Survey. Policy-specific variables in the current study, specifically as they relate to noticing and reading e-cigarette package health warnings and leaflets and experiencing undesirable events during e-cigarette use, were developed to align with the provisions of the EU TPD Article 20^9^ and respective Commission Implementing Acts^10,11^ and the reports that informed the Implementing Legislation^12,13^.

### Demographics

The survey collected data on participant demographic characteristics, including age (15–24, 25–39, 40–54 and ≥55 years), gender (male, female), and highest level of formal education completed categorized as: *low* (primary, lower pre-vocational secondary, middle pre-vocational secondary), *moderate* (secondary vocational, senior general secondary and pre-university) and *high* (higher professional and university Bachelor, university Master). Also included was whether or not they had difficulties in paying bills during the last 30 days (yes/no) as a proxy for socioeconomic status.

### E-cigarette use and frequency

Ever use of e-cigarettes was determined if respondents answered ‘yes’ to the question ‘have you ever used an e-cigarette or vaping device, even one time?’ (yes, no). Frequency of e-cigarette use was measured among those reporting ever use, by the question ‘on average, how often do you currently use e-cigarette/vaping device?’ (daily; less than daily, but at least once a week; less than weekly, but at least once a month; less than monthly; not at all). We categorized the response ‘less than daily, but at least once a week’ as weekly use and ‘less than weekly, but at least once a month’ as monthly use of e-cigarettes. We defined those reporting daily or weekly use of e-cigarettes as current e-cigarette users^21^. Additional questions on e-cigarette frequency of use were asked to those who had ever used e-cigarettes, including: ‘when was the last time you tried or used an e-cigarette/vaping device?’ (<1 year or ≥1 year); and ‘how many times have you used an e-cigarette or vaping device in your entire life?’ (≤2; 3–99; ≥100).

### E-cigarette product attributes

The survey also included questions aimed at assessing e-cigarette attributes used by consumers, including: ‘what type of e-cigarettes is your usual/current brand?’ (disposable/not refillable; replaceable pre-filled cartridges; and tank that you fill with liquids), ‘does the e-cigarette or e-liquid that you currently use/last used contain nicotine?’ (yes, no, don’t know), and ‘what flavors of e-cigarette or e-liquid have you used in the last 30 days?’ (no flavor, flavor). Those using e-cigarettes that contain nicotine were followed up with the question, ‘what is the nicotine strength of the e-cigarette/cartridge/e-liquid you used last?’ (1–8 mg/mL or 0.1–0.8%; 9–20 mg/mL or 0.9–2.0%; ≥21 mg/mL or ≥2.5%; don’t know). For those who reported using flavored e-cigarettes within the last 30 days, subsequent questions included asking about specific flavors used, such as menthol, tobacco, fruit, and chocolate/candy. Additional behavior questions included: ‘have you ever mixed different e-liquids?’ (yes, no); ‘have you ever mixed other substances (e.g. marijuana) with your e-liquid?’ (yes, no); ‘have you ever used gloves while refilling your vaping device with e-liquid’ (yes, no); ‘can you adjust power (voltage) or temperature in the e-cigarette or vaping device you currently use most?’ (no; yes, but you don’t change the settings; yes and you change the settings occasionally or regularly).

### Noticing and reading health warnings and leaflets

Noticing health warning labels on e-cigarette packaging and leaflets inside the packaging was assessed by the questions: ‘in the past 30 days, have you noticed any health warnings on packaging for e-cigarettes, cartridges, or e-liquid bottles or containers?’ (yes, no), and ‘as far as you know, is there health and product safety information contained on leaflets inside the packaging of disposable e-cigarettes, cartridges, or e-liquid?’ (yes, no). These questions were asked to respondents who reported using an e-cigarette in the past 30 days. Lastly, participants who reported having noticed warning labels or seen warning leaflets, were asked: ‘in the last 30 days, have you read any of the health warnings?’ (yes, no) and ‘have you ever read the information on the warning leaflets?’ (yes, no), respectively.

### E-cigarette-related undesirable events during use

Undesirable events related to e-cigarette use were measured by the questions: ‘in the last 6 months did you experience… “dry puff” or burnt taste with your e-cigarettes or vaping devices?’; ‘breaking or dropping the product so it no longer works?’; ‘battery overheated’; ‘battery exploded/caught on fire?’; ‘e-liquid spilling during refilling?’; and ‘e-liquid leaking during use?’ (yes, no). Additionally, to evaluate the existence of child-proof caps respondents were asked: ‘how easy or difficult would it be for a child to open the bottle or container of e-liquid?’ (it is easy for a child to open, it is difficult for a child to open such as having a child proof cap).

### Analysis

Categorical variables are presented with relative and absolute frequencies. For the comparison of proportions Pearson’s chi-squared tests (χ^2^) of independence were used. To avoid collinearity issues and owing to the sample size of some dependent variables, stepwise logistic regression analyses were applied to elucidate associations between parameters (p-value for removal was set at 0.1, and p-value for entry was set at 0.05). Stepwise regression was used because the sample size for some dependent variables was small and did not provide enough power for including together all the independent variables in the model. Specifically, for all stepwise logistic regression analyses, the following independent variables were used: age, gender, highest level of education, difficulty in paying bills, e-cig frequency of use, adjustable temperature or power capacity, and frequency of adjusting power a temperature. In addition, for the analyses with e-liquid spilling or leaking, as dependent variables, ever mixed e-liquids, volume/capacity of usual e-cig cartridge tank >2mL, child-proof e-liquid cap and warning labels that recommend using gloves were also added as independent variables. All p-values reported are two-tailed. All statistical tests and confidence intervals were corrected for the complex sample design with sampling region as strata and primary sampling unit as clusters. Analyses were conducted using SPSS statistical software (version 19.0).

## RESULTS

### E-cigarette use and frequency

A total of 6011 interviews were completed with adult smokers from six EU MS, of whom 1178 (19.6%) reported ever use of e-cigarettes (at least once in their lifetime), and of these 7.5% (88/1178) were current users (about 4.3% daily and 3.1% weekly, Table 1), with an among-EU MS variation of current use ranging from 3.1% in Spain to 12.9% in Germany (p<0.001) (Table 2). The overall prevalence of current e-cigarette use among this sample of smokers was 1.5% (88/6011). Current e-cigarette use (daily or weekly) was significantly higher among those who reported not having difficulty in paying bills in the last 30 days (7.9%, 80/1020) compared to those who reported having such difficulties (5.2%, 8/155, p<0.05) (Table 2). Among those who had ever used e-cigarettes, 15.6% had used an e-cigarette or vaping device at least 100 or more times in their lifetime (180/1179), which varied significantly by country, from 7.7% in Romania to 23.2% in Poland (p<0.01) (Table 2). Among those who had ever tried an e-cigarette, but were not currently using e-cigarettes daily, 59.4% had last tried or used an e-cigarette or vaping product one year ago or longer (662/1127) (Table 1).

**Table 1:** E-cigarette use characteristics among smokers who have ever used e-cigarettes in six European Union Member States, 2016

	*Total sample (N=1178) n (%)*
**E-cigarette frequency of use**	
*How many times have you used an e-cigarette or vaping device in your entire life?*	
2 or fewer	440 (38.1)
3–10	245 (21.2)
11–20	101 (8.7)
21–50	120 (10.4)
51–99	69 (6.0)
≥100	180 (15.6)
*On average, how often, if at all, do you currently use e-cigarette/vaping device?*	
Not at all	1000 (85.0)
Current use (daily or weekly)	88 (7.5)
Daily	51 (4.3)
Weekly (less than daily, but at least once a week)	37 (3.1)
Less than weekly (monthly and less than monthly)	89 (7.6)
*When was the last time you tried or used an e-cigarette/vaping device?^1^*	
<1 year	453 (40.6)
≥1 year	662 (59.4)
**E-cigarette product attributes**	
*What type of e-cigarettes is your usual/current brand?^2^*	
It is disposable, not refillable (non-rechargeable)	24 (14.0)
It uses replaceable pre-filled cartridges (rechargeable)	58 (33.7)
It has a tank that you fill with liquids (rechargeable)	90 (52.3)
*What flavors of e-cigarette or e-liquid have you used in the last 30 days?^3^*	
No flavor	24 (21.6)
Flavor	87 (78.4)
*Nicotine strength (mg/mL) of current/last e-liquid^4^*	
1–8	34 (36.6)
9–20	42 (43.1)
≥21	2 (2.2)
Do not know	17 (18.3)
*Ever mixed e-liquids^5^*	
No	43 (66.2)
Yes	22 (33.8)
*Does the e-cigarette or vaping device you use most frequently contain settings to adjust the power (voltage) or temperature?^6^*	
Yes, and use these settings occasionally or regularly	28 (28.8)
Yes, but do not use these settings at all	35 (36.1)
No	34 (35.1)
**E-cigarette warning labels and leaflets**	
*In the last 30 days did you…?*	
Notice warning labels on e-cig packs, cartridges, or e-liquid^7^	9 (10.2)
Read warning labels on e-cig packs, cartridges, or e-liquid^8^	6 (66.6)
Notice health warning leaflets in or on packaging^7^	21 (28.0)
Read e-cig health/safety info in or on packaging^9^	13 (61.9)
**E-cigarette undesirable events experienced**	
*In the last 6 months did you experience…?^2^*	
Dry puff or burnt taste	14 (12.7)
Broke or dropped the product so it no longer worked	9 (8.2)
Battery overheating	12 (11.0)
Battery exploding or catching on fire	2 (1.9)
Spilling during refill	12 (18.8)
Leaking during use	12 (18.5)
Nozzle on e-liquid cap easy for a child to open	14 (23.3)

Reference to those who:

1 currently do not use e-cig daily (N=1127)

2 use e-cig daily, weekly or less than weekly (N=177)

3 use e-cigs daily, weekly or monthly and used within the last 30 days (N=111)

4 use e-cigs daily, weekly or monthly and whose type of e-cig currently use most or last contains nicotine (N=95)

5 use e-cigs daily, weekly or monthly whose type of e-cig currently use most or last has a tank that you fill with liquids (N=65)

6 use e-cigs daily, weekly or monthly whose usual/current brand is rechargeable (N=97)

7 used e-cig within the last 30 days (N=90)

8 had noticed warning label

9 had seen warning leaflets.

**Table 2:** Factors associated with e-cigarette use and product attributes among cigarette smokers who have ever used e-cigarettes in six European Union member states, 2016

	*E-cigarette use*		*E-cigarette product attributes*	

	*Current e-cig users (daily or weekly) (88/1178)*	*Have used e-cigs ≥100 times in a lifetime (180/1179)*	*Use rechargeable e-cigs^1^ (148/177)*	*Use flavored e-cigs^2^ (87/111)*
	*n (%)*	*n (%)*	*n (%)*	*n (%)*
**Age (years)**				**
18–24	12 (6.9)	22 (12.6)	25 (83.3)	14 (87.5)
25–39	22 (5.3)	63 (15.5)	50 (89.3)	23 (71.9)
40–54	33 (9.2)	62 (17.5)	44 (84.6)	36 (90.0)
≥55	21 (9.4)	33 (15.1)	29 (85.3)	14 (60.9)
**Gender**				
Male	49 (7.2)	105 (15.8)	85 (88.5)	55 (85.9)
Female	39 (7.8)	75 (15.3)	63 (82.9)	32 (68.1)
**Highest level of education**				
Low	29 (7.8)	46 (12.4)	46 (85.2)	29 (78.4)
Moderate	46 (7.1)	102 (16.1)	82 (86.3)	48 (78.7)
High	13 (8.6)	30 (20.3)	20 (87.0)	10 (76.9)
**Difficulty in paying bills**	*			
Yes	8 (5.2)	21 (13.6)	18 (81.8)	8 (66.7)
No	80 (7.9)	158 (15.8)	130 (87.2)	78 (79.6)
**EU Member State**	***	**	**	
Germany	24 (12.9)	35 (18.8)	46 (85.2)	25 (80.6)
Greece	28 (12.8)	46 (21.1)	37 (92.5)	22 (71)
Hungary	8 (7.5)	12 (11.7)	13 (59.1)	10 (76.9)
Poland	9 (4.7)	42 (23.2)	23 (95.8)	11 (73.3)
Romania	12 (4.8)	19 (7.7)	20 (90.9)	12 (85.7)
Spain	7 (3.1)	26 (11.8)	9 (90.0)	7 (100.0)

* p<0.05

** p<0.01

*** p<0.001.

Referred to those who use:

1 e-cig daily, weekly or less than weekly (N=177)

2 e-cig daily, weekly or monthly in the last 30 days (N=111).

### E-cigarette product attributes

Across all six EU MS, among our sample of smokers reporting use of e-cigarettes daily or weekly (current users) as well as those reporting less than weekly use, the most frequent usual/current type of e-cigarette was a tank style (rechargeable) (52.3%), followed by replaceable pre-filled cartridges (rechargeable) (33.7%), and lastly disposable/not refillable (non-rechargeable) (14.0%) (Table 1). The percentage of those whose usual/current type of e-cigarette is rechargeable varied significantly across EU MS, ranging from 95.8% in Poland to 59.1% in Hungary (p<0.01) (Table 2). Across all MS, the majority of current e-cigarette users and those using monthly reported that their current/last e-cigarette contained nicotine (85.6%, 95/111), with users reporting using nicotine strengths of 1–8 mg/mL (36.6%), 9–20 mg/mL (43.1%), and ≥21 mg/mL (2.2%). However, 18.3% reported that they did not know the nicotine strength of their current/last e-cigarette (Table 1). Moreover, in all EU MS, the use of flavored (78.4%) e-liquids within the past 30 days was more common than unflavored e-liquids (21.6%) — the most common flavors were fruit (46.4%), followed by tobacco (27%), menthol (8.5%), and chocolate/candy (8.5%). Participants aged 40–54 years were 11.92 times more likely to use flavored e-cigarettes compared to those aged 55 and over (95% CI: 4.61–30.81) (Table 3).

**Table 3:** Factors associated with e-cigarette product attributes, as well as experiencing undesirable events during e-cigarette use in six European Union member states, 2016

	*OR (95% CI) ^+^*	*p*
Use flavors^1^ (total N=111; yes N=87, no N=24)		
*Age (years)*		
18–24	5.54 (1.04–29.66)	0.046
25–39	3.36 (1.08–10.48)	0.038
40–54	18.61 (5.67–61.14)	<0.001
≥55	1.00++	
*How often currently using e-cigs*		
Daily	8.82 (2.33–33.39)	0.003
Weekly	2.27 (0.48–10.62)	0.284
Monthly or less	1.00++	
*E-cig has adjustable power (voltage) or temperature capacity*		
No	1.00++	
Yes	5.64 (2.36–13.47)	<0.001
**Experienced dry puff^1^ (total N=110; yes N=14, no N=96)**		
*Adjustment of power (voltage) or temperature*		
No	1.00++	
Occasionally or regularly	6.01 (2.68–13.46)	<0.001
**E-liquid ever spilled during refill^2^ (total N=64; yes N=12, no N=52)**		
*E-liquid cap child-proof*		
No, difficult for a child to open	1.00++	
Yes, easy for a child to open	6.73 (2.02–22.37)	0.004
*Ever mixed e-liquids*		
No	1.00++	
Yes	8.54 (2.29–31.88)	0.004
**E-liquid ever leaked during use^2^ (total N=65; yes N=12, no N=53)**		
*Ever mixed e-liquids*		
No	1.00++	
Yes	7.78 (2.45–24.73)	0.002

+ Odds Ratio (95% Confidence Interval).

++ Indicates reference category:

1 using stepwise method with independent variables such as age, gender, highest level of education, difficulty in paying bills, e-cig frequency of use, adjustable power (voltage) or temperature capacity and frequency of adjusting power (voltage) or temperature

2 using stepwise method with independent variables such as age, gender, highest level of education, difficulty in paying bills, e-cig frequency of use, adjustable power (voltage) or temperature capacity and frequency of adjusting power (voltage) or temperature, ever mixed e-liquids, volume/capacity of usual e-cig cartridge, tank >2mL, child-proof e-liquid cap and warning labels recommend using gloves.

Among at least monthly e-cigarette users who reported use of products with a tank that the user (re-)fills with e-liquids, 33.8% reported ever mixing different e-liquids (22/65) (Table 1), 3.1% reported ever mixing other substances (i.e. marijuana) with their e-liquids (2/65) and 3.1% reported ever using gloves during e-liquid refill (2/65). Furthermore, among those whose usual/current type of e-cigarette is rechargeable, 64.9% reported that their most commonly used e-cigarette or vaping device contained settings to adjust the power (voltage) or temperature (63/97), with 28.8% reporting changing the settings either occasionally or regularly, and 36.1% had these settings but did not use them at all (35/97), while 34.1% reported not having these settings (Table 1).

### Health warning labels and leaflets

Among respondents who used e-cigarettes within the past 30 days, 10.2% ever noticed warning labels on e-cigarette packages, cartridges, or e-liquids, amongst whom 66.6% reported having read the warning labels. Further, 28.0% of participants reported having seen health and product safety information contained on leaflets, amongst whom 61.9% had read the health and safety information (Table 1).

### E-cigarette-related undesirable events during use

Among current e-cigarette daily or weekly users, as well as those reporting monthly use, the following undesirable events during e-cigarette use within the last 6 months were reported: 12.7% experienced ‘dry puff’ or burnt taste (14/110), 8.2% broke or dropped the product so it no longer worked (9/110), 11.0% experienced battery overheating (12/109), and 1.9% experienced battery exploding or catching fire (2/108) (Table 1). Among those reporting at least monthly use and whose usual/current brand has a tank that one fills with liquids, 18.8% experienced spilling during refill (12/64) and 18.5% experienced e-liquid leaking during use (12/65) within the last 6 months (Table 1). Among those who had ever spilled e-liquid during the refill process (n=12), 50.0% spilled one time (6/12), 33.3% spilled between three and ten times (4/12), and 16.7% spilled more than ten times (2/12). Lastly, 23.3% reported that the e-liquid cap was easy for a child to open (14/60) (Table 1).

### Relation between undesirable events experienced during e-cigarette use and product attributes

As noted in Table 3, participants who adjusted occasionally or regularly the power (voltage) or temperature of their e-cigarette (n=28) had significantly greater odds of ever experiencing a ‘dry puff’ compared to those whose e-cigarette had the capacity but who did not adjust the power or temperature (n=35) (OR=6.01; 95% CI: 2.68–13.46). Participants who had ever mixed different e-liquids (n=22) had 7.78 times greater odds of having e-liquid leak during use (95% CI: 2.45–24.73) and 8.54 times the odds of spilling during refill (95% CI: 2.29–31.88), compared to those who had never mixed e-liquids (n=43). Furthermore, participants whose e-liquid refill nozzle had a cap that was easy for a child to open (n=14) were 6.73 times more likely to have had e-liquid spill during refill compared to those whose e-liquid cap was difficult for a child to open (n=10) (95% CI: 2.02–22.37).

## DISCUSSION

While data on trends and predictors of e-cigarette use across the EU exist^1^, as well as data on consumer preferences for e-cigarette product attributes^22^, there is a significant research gap in the relation between e-cigarette product attributes and experiencing undesirable events during e-cigarette use. With the EU’s newly implemented TPD that took effect in May 2016, of which Article 20 sets forth provisions on e-cigarette product regulation, such as child-proof packaging and health-warning label requirements^9^, it has become more imperative to understand the impact of the TPD and to contribute to an e-cigarette regulatory science base. The current study aims to provide a baseline assessment of the characteristics and correlates of e-cigarette product attributes (both those related to EU TPD Article 20 and others warranting further research) with experiencing undesirable events during e-cigarette use and noticing health warnings and leaflets among adult smokers who use e-cigarettes from six EU MS.

Among our sample of adult cigarette smokers, 1/5 reported having ever used e-cigarettes, and of those, nearly 40% reported having used them only once or twice. Prevalence of current e-cigarette use (daily or weekly) was very low (1.5%). This can be compared to the 2017 Special Eurobarometer 458, in which 23% of current smokers reported having tried e-cigarettes once or twice and 4% of current smokers reported currently using e-cigarettes across all 28 EU MS^23^. It is interesting to note that the six EU MS in the current study were among the lowest users of e-cigarettes reported in the 2017 Special Eurobarometer 458 (Germany 2%, Greece 3%, Hungary 1%, Poland 1%, Romania 0%, Spain 1%)^23^. The majority of ever users of e-cigarettes in our sample of smokers reported using them infrequently, with most on a monthly or less basis, having used e-cigarettes less than 100 times in their lifetime, and having used their last e-cigarette one year or more ago. Not having difficulty with paying bills on time was associated with greater daily e-cigarette current use compared to those who had difficulty paying bills, consistent with other studies showing that among smokers, those with higher socioeconomic status are more likely to use e-cigarettes^24^. The low levels of current e-cigarette use among smokers in our sample may be partly explained by the very high smoking rates in the EU (26% EU MS average), with the six EU MS included in the current study having some of the highest smoking rates in the EU (Germany 25%, Greece 37%, Hungary 27%, Poland 30%, Romania 28%, and Spain 28%)^23^. This compares with other populations with lower smoking rates such as the US (15.1% in 2016)^25^ where e-cigarette use among smokers is also more prevalent, with 37% ever use and 10.6% current use (>1 time per day in last 30 days among current smokers)^26^.

Our study further provides insight into the type of e-cigarette product attributes used by smokers who use e-cigarettes in the EU. We found that respondents overall and across all six EU MS reported use of flavored e-cigarettes, with the most common flavors identified as fruit, tobacco and menthol, which varied significantly by age group. This is consistent with other studies identifying e-cigarette consumer use of flavors, with tobacco, menthol and fruit ranking among the top flavors^22,27,28^. However, in contrast to previous research^27^, we found that young adults (18–24 years) were less likely to prefer flavors than those who were older (25–54 years). This discrepancy may be explained by the fact that our sample only included smokers and we characterized ‘tobacco’ as a flavor, which previous research has suggested is more common among the older adults^29^, while sweet-flavored e-cigarette products have been found to be more popular among younger adults^30^, although still favored generally by adults^22,28^. Our findings on the high prevalence of flavored e-cigarette products across our sample raise concerns given the mounting research on the toxicity profile of flavors and impact on reduced harm perceptions, an area needing further examination^12,17^. With only a handful of studies examining e-cigarette flavors in European populations^31,32^, this study is an important contribution for characterizing the use of e-cigarette flavors within the EU, where potential regulations in this area are within the scope of the EC^9^.

Furthermore, we found that most smokers used e-cigarette products containing nicotine, supporting the findings of previous studies^22^. However, 1 in 5 respondents whose current brand contained nicotine did not know the nicotine strength of their current e-cigarette brand, which may increase the risk of inadvertent high nicotine exposure^22^. This finding is further confounded by results from other studies indicating discrepancies between reported and measured nicotine content in e-liquid products^33^. Use of rechargeable e-cigarettes of tank style was more likely among our sample and mixing e-liquids was a common practice, consistent with previous studies due to consumers’ ability to mix their own e-liquids to customize flavors and strength, an area in which there is less regulatory control or consumer quality control oversight^22,34^.

Our study additionally indicates that prior to EU TPD full implementation, the majority of respondents reported not noticing or reading e-cigarette health warning labels on outside packaging or pamphlet inserts. It will be important to monitor the impact of health warning parameters after full TPD implementation, in which health warning labels and leaflets will be required and standardized^9^. However, it may also be necessary to implement strategies for increasing the noticing and reading of health warnings. The range of e-cigarette product and packaging designs poses a challenge for how e-cigarette warning labels may be noticed. There is also little research on what type of health warnings are most effective for e-cigarettes^18^.

We further identified frequencies and correlates of undesirable events related to product design parameters, many of which the EU TPD aims to address. Major undesirable events, such as the battery exploding or overheating, were uncommon but not negligible. If such events persist after full EU TPD implementation, which aims at more standardized manufacturing processes overall and inclusion of leaflets on proper use, it may be necessary to consider other safety measures such as battery design requirements and battery testing standards. In contrast, a lack of child proof e-liquid caps and e-liquids spilling during use and refill were considerable, which is concerning given the risks associated with skin exposure and ingestion of nicotine-containing liquids^12,35^. In light of the EU TPD requiring child-resistant packaging and protection against refilling leakage, these findings further support its implementation as well as the monitoring of design features once fully adopted by MS, as child resistant packaging is a design parameter that can be easily addressed^9,36^. Further, those who adjusted the power (voltage) or temperature of their e-cigarettes were more likely to experience problems with ‘dry puff’ or burnt taste. Some studies suggest that refillable e-cigarettes with the ability to adjust the voltage may increase the risk of exposure to toxicants, such as carbonyls and aldehydes at high voltage settings, particularly during ‘dry puff’ conditions. Thus the adjustable voltage is an attribute that leads potentially to the incorrect consumer use of the e-cigarette^15,36–38^. Our results indicate how product attributes currently monitored by the EU TPD (i.e. lack of child-proof caps), as well additional attributes (i.e. mixing e-liquids), may increase user experience of undesirable events during e-cigarette use (i.e. spilling liquid).

Our findings offer new insights to e-cigarette attributes and use for a population in a geographic area for which the literature is sparse, with previous studies on e-cigarette product attributes primarily conducted in the United States and Canada^22^, which have very different policy environments, and patterns of cigarette and e-cigarette use, from those in Europe^39^. More noteworthy is that our study took place within the geographic location for which the EU TPD applies, thereby providing a launching pad for evaluating the impact of the implementation of the regulatory standards of e-cigarettes in the EU TPD Article 20. Monitoring how polices aimed at e-cigarette product attributes impact consumers have vast policy implications that go beyond the EU, particularly for countries that have not established or not yet implemented such regulations. For instance, the US Food and Drug Administration (FDA) Center for Tobacco Products was granted regulatory authority over e-cigarettes in 2016^40^, however with its new regulatory shift focused on nicotine and addiction, the FDA has extended many regulatory deadlines for e-cigarettes until 2022^41^, with very few provisions related to product design characteristics or attributes. Further, while increasingly more countries are regulating e-cigarettes and the Conference of the Parties to the WHO FCTC have identified general regulatory strategies for e-cigarettes, the approaches are primarily constrained to policies on minimum-age-of-purchase, vape-free public places, and advertising, with limited focus and guidance on regulation related to product attributes and health warnings^18,42^.

### Strengths and limitations

This study offers new data on characteristics of e-cigarette attributes and fills in some important research gaps in experiencing undesirable events during e-cigarette use, particularly as they relate to the EU TPD. However, there are some limitations of the present study that must be considered when interpreting results.

Firstly, our study sample only included cigarette smokers who also reported e-cigarette use (dual users), precluding a comparator group of exclusive e-cigarette users who may differ in experiencing undesirable events as a function of user experience. Recent studies among European populations suggest that exclusive e-cigarette users are more likely to use e-cigarettes more frequently and on a daily basis compared to dual users^24^. As such, these two populations of e-cigarette users may differ on many aspects related to e-cigarette product attributes and in experiencing undesirable events during use. Therefore, the current study may not be generalizable to exclusive e-cigarette users but is an area that deserves further evaluation. It should be noted that the current study controlled for frequency of e-cigarette use. While there are limitations with the exclusion of non-smokers, adult cigarette smokers who also use e-cigarettes are a population of particular interest for elucidating how the EU TPD in its entirety will impact both cigarette and e-cigarette behaviors over time.

Secondly, despite starting with a large, nationally representative sample of over 6000 smokers, the sample size used in the present analyses was small, and caution should be exercised in the generalization of these findings. The small sample size also limited the number of covariates that could be assessed in the regression analyses, such as frequency of cigarette use and quitting behaviors, variables which may impact on the outcome variables examined. Nevertheless, while prevalence of dual use was low within the studied sample, within the context of the imminent EU TPD, it is particularly important to have a baseline in order to monitor and evaluate how policy changes impact on these issues over time.

Lastly, our study relied on self-reported data that are cross-sectional. Thus, findings may be subject to respondent bias, and conclusions cannot be made on the direction of associations or causal inferences.

## CONCLUSIONS

Within this cross-sectional study of adult cigarette smokers across six EU MS conducted in 2016 prior to full implementation of the EU TPD Article 20, we examined factors associated with e-cigarette attributes and identified correlates of undesirable events during e-cigarette use. We found that the most used e-cigarette products, among our sample of smokers, were refillable products that have a tank that can be filled with liquids, as well as e-liquids that contained nicotine and were flavored. Mixing e-liquids, a popular e-cigarette practice among respondents, was correlated with spilling liquid during refill and leaking during use. Handling e-liquid vials with caps described as being easy for a child to open were also associated with greater odds of spilling during refill. Moreover, adjusting e-cigarette’s power (voltage) or temperature was associated with experiencing ‘dry puff’ or burnt taste. While these findings provide a comprehensive baseline assessment, longitudinal research is needed to better understand how changes to e-cigarette product parameters with Article 20 TPD implementation, such as health warning labels and child-resistant packaging, will impact on e-cigarette patterns and experiencing undesirable events during use, an aspect that will be addressed within the context of this Horizon 2020 project.
